# Immunophenotype Heterogeneity in Nasal Glomangiopericytoma

**DOI:** 10.1155/2015/308743

**Published:** 2015-08-17

**Authors:** Adriana Handra-Luca, Zakaria Y. Abd Elmageed, Christina Magkou, Marick Lae

**Affiliations:** ^1^APHP, Université Paris Nord Sorbonne Cité, 93009 Bobigny, France; ^2^Tulane Medical School, New Orleans, LA 70118, USA; ^3^Evaggelismos General Hospital, 10675 Athens, Greece; ^4^Institut Curie, 75005 Paris, France

## Abstract

Nasal glomangiopericytoma is rare. The immunophenotype is heterogeneous, more frequently smooth-muscle-actin and CD34-positive. We report expression patterns for several vascular-related proteins such as CD99, CD146, Bcl2, and WT1 as well as for treatment-related proteins such as mTOR and EGFR in a nasal glomangiopericytoma. The patient (woman, 86 years) presented with a left nasal tumefaction. The resected specimen (1.5-cm) showed a glomangiopericytoma. Tumor cells expressed smooth-muscle-actin, CD31, CD34, and progesterone receptor. They also expressed the vascular-cell-related proteins Bcl2, CD99, CD146, and WT1, as well as mTOR and EGFR. Nasal glomangiopericytomas show immunohistochemical heterogeneity for vascular-related markers, suggesting a possible extensive pericytic differentiation. The expression of potential targets for drug treatments such as mTOR and EGFR may impact on the clinical follow-up of these tumors occurring at advanced ages, which may require complex surgery.

## 1. Introduction

Nasal glomangiopericytoma is rare tumors, known to show diffuse expression of smooth-muscle-actin and focal expression of CD34. Bcl2 and CD99 are reported as negative in the few tested nasal tumors [1–4]. Here we report expression patterns for several vascular-related proteins such as CD99, CD146, Bcl2, and WT1 as well as for treatment-related proteins mTOR and EGFR in a nasal glomangiopericytoma.

## 2. Case Report

**Figure 1:**
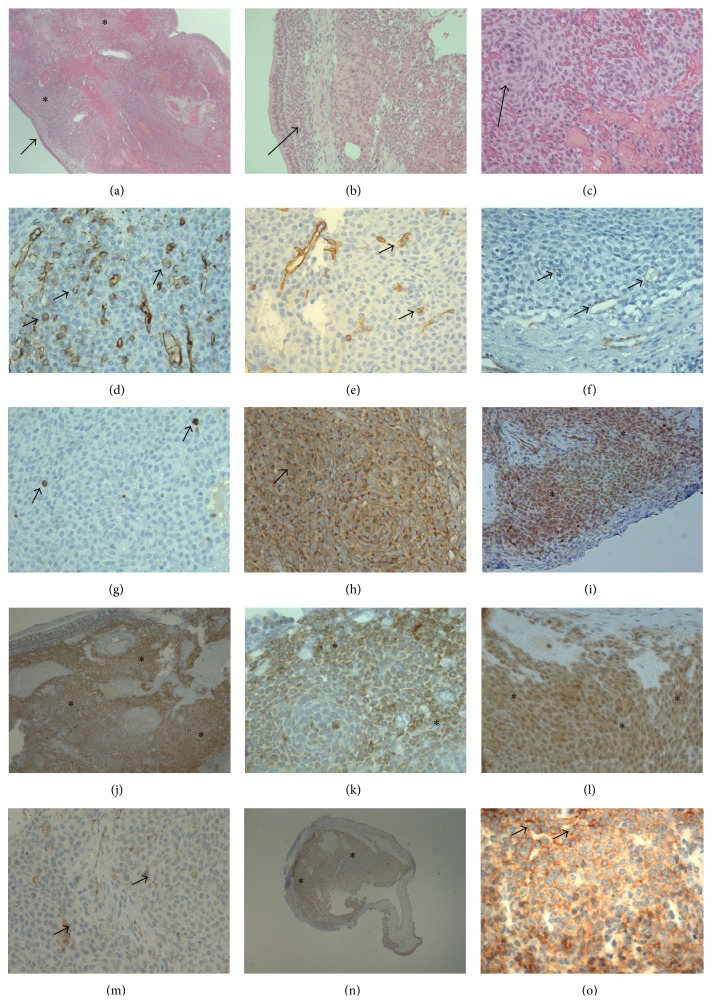
(a)–(c) Histopathological section of the biopsy showing the tumor as located in the nasal mucosa ((a) asterisks for the tumor tissue, arrow for mucosa). (b) Focal collection of submucosal tumor cells (arrow). (c) Occasional whirling pattern of tumor cells (arrow). ((a)–(c) Hematoxylin and eosin stain.) (d)–(n) Tumor cells expressing CD31 (d) and CD34 (e), showing missing expression of D2-40 ((f) arrows for rare intratumor positive vessels), expressing nuclear Ki67 ((g) arrows), CD99 ((h) dot-like cytoplasmic expression in some tumor cells/arrow), CD146 ((i) asterisks for positive tumor cells), and Bcl2 ((j)-(k) asterisks in the positive zones, some of them at the periphery of negative whirling zones), as well as progesterone receptor ((l) asterisks), WT1 ((m) arrows), and expressing moderately and diffusely mTOR ((n) asterisks) and membrane and cytoplasmic EGFR ((o) arrows). Magnification ×25 ((a), (n)), ×50 ((b), (j)), ×200 (i), and ×400 ((c), (d), (e), (f), (g), (h), (k), (l), (m), (o)).

The patient (woman, 86 years) complained of nasal tumefaction. The medical history included left colon cancer (32 years previously) and arterial hypertension. On rhinoscopy there was a left nasal, septal, polypoid lesion. This lesion was resected. The specimen was fixed in formalin 10% and then included in paraffin. Tissue sections (3 *µ*m thick) were stained with hematoxylin and eosin, reticulin, PAS, and reticulin. Tissue sections were also used for the immunohistochemistry techniques (Benchmark IHC/ISH automate) for the following antibodies: Bcl-2 (Dako clone 124), CD3 (Dako, polyclonal), CD20 (Ventana/Roche clone L-26), CD31 (Dako clone JC70A), CD34 (Dako clone QBEnd-10), CD56 (Novocastra clone 1B6), CD99 (Dako clone 12E7), CD117 (Ventana/Roche, clone T595), CD133 (Cell Signaling Technology clone C24B9), chromogranin (Dako clone DAK-A3), desmin (Dako clone D33), EMA (Dako clone E29), estrogen receptor (Novocastra clone 6F11), HER2 (Ventana/Roche clone 4B5), HMB45 (Dako clone HMB45), Ki67 (Ventana/Roche clone 4A4), melan A (Dako clone A103), mTOR (Cell Signalling Technology clone 49F9), progesterone receptor (Novocastra clone 16), Ros1 (Cell Signaling Technology clone D4D6), smooth-muscle-actin (Dako, clone 1A4), synaptophysin (Ventana/Roche clone SP11), S100 protein (Dako, polyclonal), and WT1 (Cell Marque clone 6F-H2). For the anti-CD146 antibody (Invitrogen/Life Technologies, polyclonal), the immunohistochemistry technique was performed manually [5] while for the anti-EGFR antibody (Monosan, MONX10173) a Dako automate was used. The specimen, measuring 1.5 × 1 cm showed on microscopy a diffuse small-cell proliferation in the submucosa associated with a subepithelial rim of tumor cells (Figure 1). The overlying mucosa was focally eroded. Tumor cells were round and spindled and disposed focally in a whirling pattern. Cellular atypia was mild. Stroma was sparse, mainly surrounding stromal vessels, and showed hemorrhage and hemosiderin. Focally, tumor cells were in contact with the vascular endothelium or lined directly vascular spaces. On immunohistochemistry very sparse tumor cells expressed smooth-muscle-actin (mainly lining directly vascular spaces), CD31 and CD34. Bcl2 was expressed heterogeneously, less intensely or lacking in whirling zones, while CD99 and CD146 were expressed diffusely. For CD99 a cytoplasmic dot-like expression pattern was seen. Progesterone-receptor expression was diffuse. Smooth-muscle-actin, CD31, CD34, CD99, and CD146 were also expressed in some vascular spaces or stromal vessels. mTOR was expressed in tumor cell nuclei and cytoplasm. WT1 was expressed in the cytoplasm of rare tumor cells. There was no immunohistochemical Stat6 nuclear translocation. EGFR showed multifocal membrane and cytoplasmic staining. The Ki67 index was 5%. Ros1, p63 protein, CD3, CD20, melan A, HMB45, cytokeratin AE1/3, chromogranin, synaptophysin, CD56, S100 protein, D2.40, desmin, HER2, and CD133 immunohistochemistries were negative in tumor cells. There was no EWS-type rearrangement in tumor cells by FISH analysis. The diagnosis of nasal glomangiopericytoma was proposed. At 6 months after resection, the patient was doing well, without recurrence.

## 3. Discussion

Here we report immunohistochemical heterogeneity for vascular-related markers in a case of nasal glomangiopericytoma. The immunophenotype of tumor cells in the present case consisting in Bcl2, CD99, and progesterone-receptor positivity and with very focal expression of smooth-muscle-actin, CD31, and CD34 was suggestive for a meningeal hemangiopericytoma rather than for an extracranial hemangiopericytoma/solitary fibrous tumor-type lesion [6]. However, no meningeal tumor nor Stat6 translocation was identified in our case [6, 7]. Of note would be the extensive expression of CD146 in the cytoplasm of most tumor cells as opposed to that of other vascular markers such as CD31, CD34, or smooth-muscle-actin. Whether this is related to predominant pericytic differentiation remains to be further studied. We have also observed nuclear mTOR expression in tumor cells, feature previously reported in solitary fibrous tumors, the same as EGFR expression abnormalities [8, 9], potential targets for orienting drug treatments for nasal glomangiopericytomas, especially when occurring at advanced ages or requiring complex surgery.
